# A predictive model for multimodal rehabilitation efficacy in post-stroke patients with lower limb motor impairment

**DOI:** 10.3389/fneur.2025.1714424

**Published:** 2025-12-11

**Authors:** Bing Cao, Jincheng Zhang

**Affiliations:** 1 Neurology Department, Tianjin Second People's Hospital, Tianjin, China; 2 Jiamusi College, Heilongjiang University of Chinese Medicine, Jiamusi, China

**Keywords:** stroke, lower limb motor impairment, functional electrical stimulation, mirror therapy, prediction model

## Abstract

**Objective:**

To explore the feasibility and clinical value of constructing a therapeutic efficacy prediction model for patients with lower limb motor dysfunction after stroke who received conventional treatment combined with functional electrical stimulation (FES) mirror therapy training, based on age, baseline National Institutes of Health Stroke Scale (NIHSS) score, baseline FMA score, FES stimulation intensity, FES stimulation frequency, and mirror therapy training duration.

**Methods:**

A total of 510 patients with lower limb motor dysfunction after stroke admitted to the hospital from January 2022 to October 2024 were selected and divided into a training set (*n* = 357) and a validation set (*n* = 153) at a ratio of 7:3. The clinical data of the patients were collected, and the FES stimulation parameters and mirror therapy training data were recorded. The modified Fugl-Meyer Motor Assessment Scale (FMA) was used to evaluate the therapeutic efficacy (effective was defined as an improvement of FMA score ≥ 15 points). Independent risk factors were screened by univariate and multivariate Logistic regression, a Nomogram model was constructed, and its efficacy was evaluated and verified.

**Results:**

The effective treatment rate was 65.83% (235/357) in the training set and 64.05% (98/153) in the validation set. Multivariate regression showed that age, baseline NIHSS score, baseline FMA score, FES stimulation intensity, FES stimulation frequency, and mirror therapy training duration were independent influencing factors (All *p* < 0.05). The C-indices of the Nomogram model in the training set and the validation set were 0.792 and 0.778 respectively, and the AUCs were 0.789 (95% CI: 0.728–0.851) and 0.774 (95% CI: 0.681–0.867) respectively. The sensitivities and specificities were 0.779, 0.700 and 0.714, 0.738, respectively. The calibration curves showed good consistency between the predicted values and the actual values, and the *P*-values of the Hosmer-Lemeshow test were 0.866 and 0.442, respectively.

**Conclusion:**

The Nomogram model constructed based on the above indicators can effectively predict the therapeutic efficacy of patients with lower limb motor dysfunction after stroke, providing a basis for clinical individualized intervention.

## Introduction

Stroke was one of the leading causes of death and disability globally. In China, approximately 2.4 million new stroke cases are reported annually, with 70–80% of survivors experiencing limb motor impairment. Lower limb functional impairment affects the ability to stand, walk, and overall quality of life (1, 2). Epidemiological surveys have shown that 63% of stroke patients have a lower limb Fugl-Meyer Assessment (FMA) score ≤20 points, and 45% of patients remain unable to complete independent walking tasks 6 months after onset (3, 4). Functional electrical stimulation (FES) stimulates *α*-motor neurons through electric current to induce rhythmic muscle contractions. This can be applied to enhance the strength of the tibialis anterior muscle and improve the dorsiflexion function of the ankle joint. Mirror therapy activates the primary motor cortex and secondary motor areas through visual feedback, promoting the reconstruction of motor imagery of the affected limb. The clinical efficacy rate of combining these two interventions with conventional rehabilitation therapy ranges from 58 to 72% (5, 6).

However, significant differences in treatment efficacy are observed among patients in clinical practice. Under the same treatment regimen, young patients showed a 23–35% greater improvement in FMA scores compared to elderly patients, and the effective rate of patients with mild baseline neurological deficits is 2.1 times that of those with severe deficits (7). Currently, the evaluation of treatment efficacy mainly relies on post-treatment functional scores, and there is a lack of accurate pretreatment prediction tools, making it difficult to implement individualized interventions at an early stage in clinical practice (8, 9). Although some studies have indicated that the baseline National Institutes of Health Stroke Scale (NIHSS) score is related to motor function recovery, key variables such as FES stimulation parameters and the dosage of mirror therapy have not been integrated (10, 11).

With the advancement of the precision rehabilitation concept, developing an efficacy prediction model based on multidimensional indicators holds significant clinical value for identifying high-risk patients and optimizing treatment regimens (12). This study intends to establish a nomogram prediction model by analyzing the interaction among age, baseline neurological function, and treatment parameters, providing decision-making support for the precision rehabilitation of lower limb dysfunction after stroke.

## Materials and methods

### Study subjects

A total of 510 patients with lower-limb motor dysfunction due to stroke who were admitted to our hospital from January 2022 to October 2024 were selected as research subjects. Inclusion criteria were as follows: (1) The patients were diagnosed with stroke by cranial CT or MRI and had lower-limb motor dysfunction (FMA lower-limb score ≤20 points). (2) The course of the disease was ≤ 6 months, (3) The age ranged from 18 to 80 years old. (4) The patients were conscious and able to cooperate with rehabilitation training. (5) The patients signed the informed consent form. Exclusion criteria were as follows: (1) The patients had combined severe dysfunction of vital organs such as the heart, lungs, liver, and kidneys. (2) The patients had contraindications for FES treatment (such as skin damage, a history of epilepsy, etc.). (3) The patients had other neurological or mental diseases. (4) The patients were pregnant or lactating women. The patients were randomly divided into a training set (*n* = 357) and a validation set (*n* = 153) at a ratio of 7:3 using the complete randomization method (13). This study was approved by the hospital ethics committee.

### Data collection

General clinical data of the patients were collected, including age, gender, BMI, stroke location, stroke type (Ischemic/Hemorrhagic), disease duration, history of hypertension, history of diabetes, history of hyperlipidemia, and anti-platelet medication usage. Cranial CT or MRI (used for initial diagnosis of stroke, including ischemic and hemorrhagic subtypes) was further used to measure lesion size (infarct size for ischemic stroke, hematoma size for hemorrhagic stroke, all classified into three categories: <5 cm^3^, 5–10 cm^3^, and >10 cm^3^) and record lesion location (stratified as basal ganglia, cortex, brainstem, or multiple regions). FES treatment parameters were recorded: stimulation intensity (mA) and stimulation frequency (Hz), as well as mirror therapy training data: duration of each training session (min) and number of daily training sessions. The treatment cycle was 8 weeks. After the treatment, the lower limb motor function was evaluated using the Modified Fugl-Meyer Assessment Scale (FMA) lower limb subscale (including 17 items covering hip, knee, ankle movement, and balance) to evaluate therapeutic efficacy; effective was defined as an FMA lower limb subscale improvement ≥ 15 points (14). Outcome assessors responsible for FMA evaluation were 2 senior physiotherapists with>5 years of clinical experience in stroke rehabilitation. They were blinded to two key pieces of information: (1) patient grouping (training set vs. validation set) and (2) treatment parameters (FES stimulation intensity, mirror therapy duration) to avoid assessment bias. Inter-assessor reliability was pre-tested on 30 randomly selected patients, with an intraclass correlation coefficient (ICC) of 0.92, indicating good consistency between assessors.

### Treatment protocol

All patients received conventional rehabilitation treatment for stroke, including exercise therapy, occupational therapy, and physical factor therapy. On this basis, FES and mirror therapy were combined:

FES treatment: A dual-channel functional electrical stimulator was used. Electrode pads were placed on the quadriceps femoris and tibialis anterior muscles of the affected side, respectively. The stimulation intensity was set at the maximum tolerable contraction intensity for the patient (ranging from 10–40 mA), and the stimulation frequency was 20–50 Hz. Each session lasted for 20 min, once a day.

Mirror therapy: A mirror box device was adopted. The affected limb was placed behind the mirror box, and the healthy limb was placed in front. Through mirror reflection, the patient was provided with visual feedback of normal movement of the affected side. Each training session lasted for 30 min, 1–2 times a day.

### Sample size calculation

Based on previous studies (12), assuming a medium effect size (OR = 1.2) for the primary predictor (age) on treatment efficacy, significance level *α* = 0.05, and power *β* = 0.8, the required minimum sample size was calculated as 400 using GPower 3.1 software; our final sample size of 510 (357 in training set, 153 in validation set) met and exceeded this requirement.

### Statistical analysis

Data analysis was conducted using SPSS 26.0 and R 4.2.1 software. When the measurement data conforms to the normal distribution, the measurement data were presented as mean ± standard deviation, and the independent-samples t-test was used for comparisons between groups. M (Q1, Q3) is used when it does not conform to the normal distribution, and Mann–Whitney U test is used for comparison between groups. Count data were presented as the number of cases and percentages (*n*, %), and the *χ*^2^ test or Fisher exact method was used for comparisons between groups. Univariate analysis and multivariate Logistic regression analysis were used to screen for independent influencing factors, and their odds ratios (OR) and 95% confidence intervals (CI) were calculated. A nomogram model was constructed based on the independent influencing factors. The predictive efficacy of the model was evaluated by the receiver operating characteristic curve (ROC), and the area under the curve (AUC) and 95% CI were calculated. The calibration curve and Hosmer-Lemeshow test were used to evaluate the consistency between the predicted values and the actual values. Additionally, Bootstrap validation (1,000 resamples from the training set) was performed to assess the model’s robustness. Decision curve analysis (DCA) was used to evaluate the clinical application value of the nomogram by calculating the net benefit at different threshold probabilities. A *p* value < 0.05 was considered statistically significant.

## Results

### Comparison of baseline data of patients with lower-limb motor dysfunction after stroke between the training set and the validation set

A total of 510 patients with lower limb motor dysfunction after stroke were included and divided into a training set (*n* = 357) and a validation set (*n* = 153). There were no significant differences in indicators such as age, gender, BMI, stroke location, stroke type, disease duration, baseline NIHSS score, and baseline FMA score between the training set and the validation set (All *p* > 0.05), indicating comparability (Table 1).

**Table 1:** Comparison of baseline data of patients with lower-limb motor dysfunction after stroke between the training set and the validation set.

Indicators	Training set (*n* = 357)	Validation set (*n* = 153)	*t/χ^2^*	*P*
Age (years)	61.69 ± 8.22	61.28 ± 8.09	0.521	0.603
Sex (Male/Female)	212/145 (59.38/40.62)	80/73 (52.29/47.71)	2.204	0.137
BMI (kg/m^2^)	24.57 ± 3.31	24.53 ± 3.45	0.124	0.902
Stroke location (Cortex/Subcortex)	185/172 (51.82/48.18)	79/74 (51.63/48.37)	0.001	0.969
Lesion size (cm^3^) (<5/5–10/>10)	185/142/30 (51.82/39.78/8.40)	80/62/11 (52.29/40.52/7.19)	0.215	0.898
Stroke type (ischemic/hemorrhagic)	315/42 (88.37/11.63)	130/23 (84.97/15.03)	1.029	0.311
Disease duration (days)	46.39 ± 12.76	47.65 ± 11.85	1.044	0.297
Baseline NIHSS score	12.79 ± 3.89	12.34 ± 3.75	1.211	0.227
Baseline FMA score	13.41 ± 3.59	13.12 ± 3.56	0.838	0.402
History of hypertension (yes/no)	210/147 (58.82/41.18)	100/53 (65.36/34.64)	1.919	0.166
History of diabetes (yes/no)	96/261 (26.89/73.11)	40/113 (26.14/73.56)	0.031	0.861
History of hyperlipidemia (yes/no)	137/220 (38.38/61.62)	50/103 (32.68/67.32)	1.496	0.221
Anti-platelet medication use (yes/no)	325/32 (91.04/8.96)	140/13 (91.50/8.50)	0.029	0.865
FES stimulation intensity (mA)	25.67 ± 5.96	24.86 ± 6.01	1.403	0.161
FES stimulation frequency (Hz)	37.27 ± 6.05	36.52 ± 6.03	1.284	0.199
Mirror therapy training duration (min/session)	31.08 ± 4.98	30.26 ± 5.53	1.648	0.101
Daily frequency of mirror therapy (times)	1.80 ± 0.41	1.81 ± 0.52	0.232	0.817

### Univariate analysis of influencing factors for treatment efficacy in patients with lower-limb motor dysfunction after stroke

In the training set, there were 235 cases (65.83%) in the treatment-effective group and 122 cases (34.17%) in the ineffective group. Univariate analysis showed that there were statistically significant differences in age, baseline NIHSS score, baseline FMA score, FES stimulation intensity, FES stimulation frequency, and mirror therapy training duration between the effective group and the ineffective group (All *p* < 0.05) (Table 2). Among the effective group, the main improved domains of the FMA lower limb subscale were ankle dorsiflexion (mean increase: 4.2 ± 1.5 points) and knee extension (mean increase: 3.8 ± 1.2 points), which accounted for 62% of the total FMA lower limb subscale improvement.

**Table 2:** Univariate analysis of influencing factors for treatment efficacy in patients with lower-limb motor dysfunction due to stroke in the training set.

Indicators	Effective group (*n* = 235)	Ineffective group (*n* = 122)	*t/χ^2^*	*P*
Age (years)	60.51 ± 7.89	63.98 ± 8.42	3.851	0.001
Sex (male/female)	142/93 (60.43/39.57)	70/52 (57.38/42.62)	0.309	0.578
BMI (kg/m^2^)	24.81 ± 3.11	24.12 ± 3.61	1.881	0.061
Stroke location (cortex/subcortex)	115/120 (48.94/51.06)	70/52 (57.38/42.62)	2.292	0.131
Lesion size (cm^3^) (<5/5–10/>10)	125/92/18 (53.19/39.15/7.66)	60/50/12 (49.18/40.98/9.84)	0.892	0.640
Stroke type (ischemic/hemorrhagic)	210/25 (89.36/10.64)	105/17 (86.07/13.93)	0.841	0.359
Disease duration (days)	45.61 ± 12.32	47.91 ± 13.51	1.618	0.107
Baseline NIHSS score	12.31 ± 3.62	13.71 ± 4.23	3.268	0.001
Baseline FMA score	14.21 ± 3.52	11.86 ± 3.21	6.162	0.001
History of hypertension (yes/no)	132/103 (56.17/43.83)	78/44 (63.93/36.07)	1.999	0.157
History of diabetes (yes/no)	56/179 (23.83/76.17)	40/82 (32.79/67.21)	3.277	0.071
History of hyperlipidemia (yes/no)	85/150 (36.17/63.83)	52/70 (42.62/57.38)	1.414	0.234
Anti-platelet medication use (yes/no)	215/20 (91.49/8.51)	110/12 (90.16/9.84)	0.173	0.678
FES stimulation intensity (mA)	26.21 ± 5.81	24.62 ± 6.13	2.407	0.017
FES stimulation frequency (Hz)	38.51 ± 6.21	34.88 ± 5.83	5.348	0.001
Mirror therapy training duration (min/session)	31.52 ± 4.86	30.23 ± 5.13	2.334	0.021
Daily frequency of mirror therapy (times)	1.81 ± 0.39	1.78 ± 0.45	0.653	0.514

### Multivariate logistic regression analysis of influencing factors for treatment efficacy in patients with lower-limb motor dysfunction after stroke

The treatment efficacy was taken as the dependent variable, and the indicators with *p* < 0.05 in the univariate analysis were included as covariates in the multivariate Logistic regression model. The results showed that age, baseline NIHSS score, baseline FMA score, FES stimulation intensity, FES stimulation frequency, and mirror therapy training duration were significantly associated with treatment efficacy in patients with lower-limb motor dysfunction after stroke (All *p* < 0.05) (Table 3).

**Table 3:** Multivariate logistic regression analysis of patients with lower limb motor dysfunction after stroke.

Items	B	SE	Wald	*P*	OR	95% CI
Age	−0.046	0.017	7.631	0.006	0.955	0.924–0.987
Baseline NIHSS score	−0.096	0.034	8.127	0.004	0.908	0.850–0.970
Baseline FMA score	0.192	0.039	23.841	0.001	1.212	1.122–1.309
FES stimulation intensity	0.060	0.022	7.342	0.007	1.061	1.017–1.108
FES stimulation frequency (Hz)	0.118	0.023	25.466	0.001	1.125	1.075–1.177
Mirror therapy training duration	0.053	0.026	4.208	0.040	1.054	1.002–1.109

### Construction and validation of the nomogram prediction model for therapeutic efficacy in patients with lower-limb motor dysfunction after stroke

**Figure 1:**
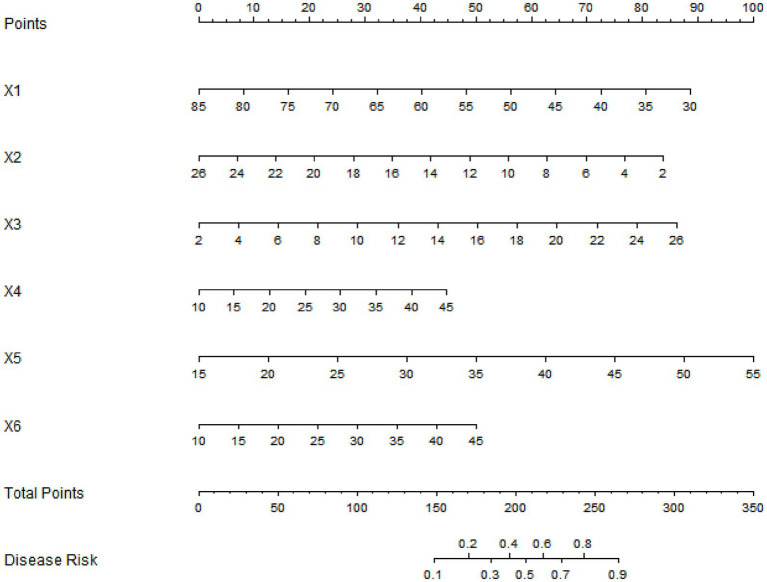
Nomogram prediction model for the therapeutic efficacy of patients with lower-limb motor dysfunction after stroke. x1: age; x2: baseline NIHSS score; x3: baseline FMA score; x4: FES stimulation intensity; x5: FES stimulation frequency; x6: training duration of mirror therapy.

A nomogram model was constructed based on the independent risk factors identified through multivariate Logistic regression. In the model, corresponding scores were assigned to each variable according to the regression coefficients, and the total score corresponded to the predicted probability of effective treatment (Figure 1).

**Figure 2:**
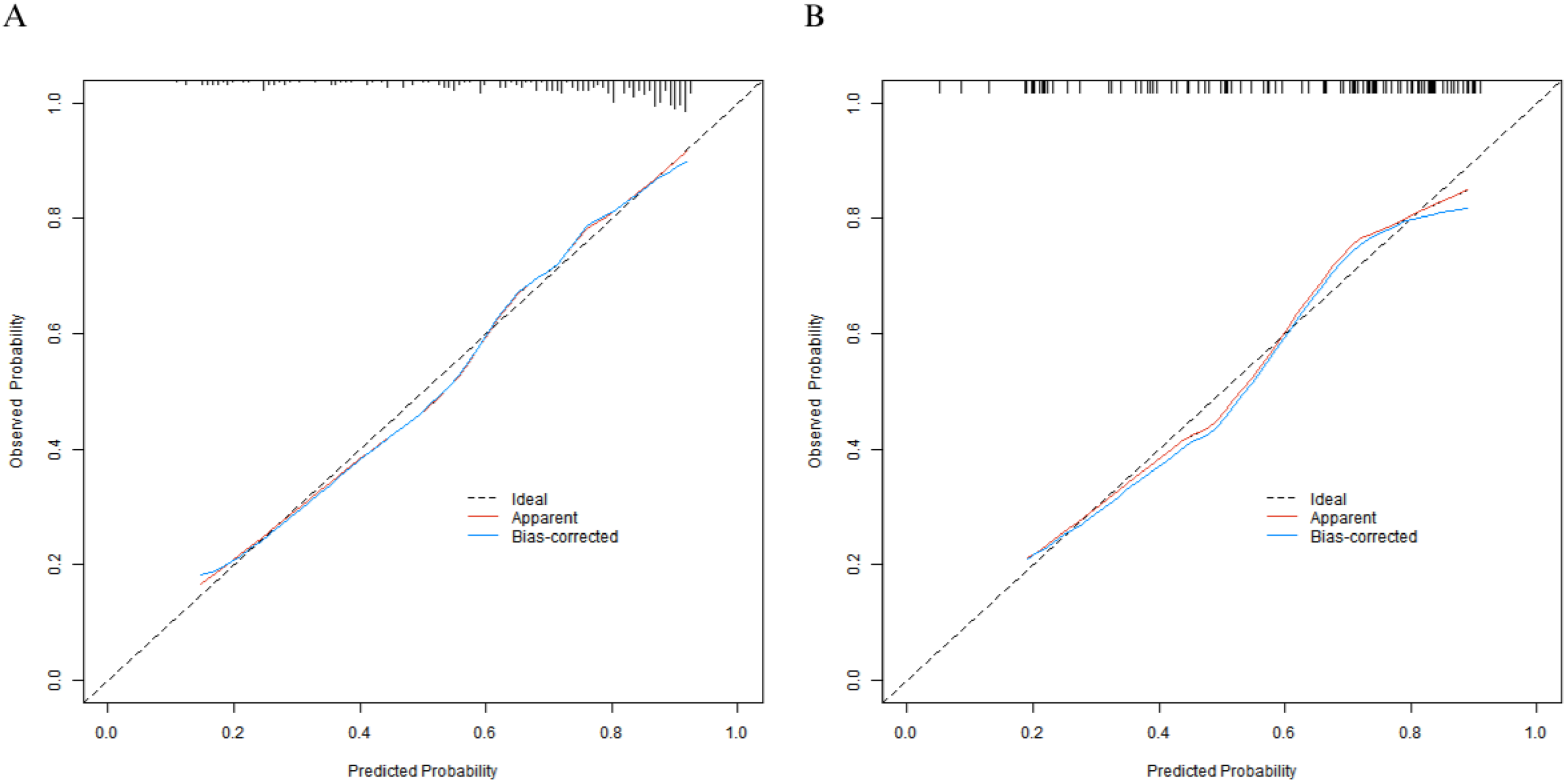
Calibration curves of patients with lower-limb motor dysfunction after stroke in the training set **(A)** and the validation set **(B)**.

**Figure 3:**
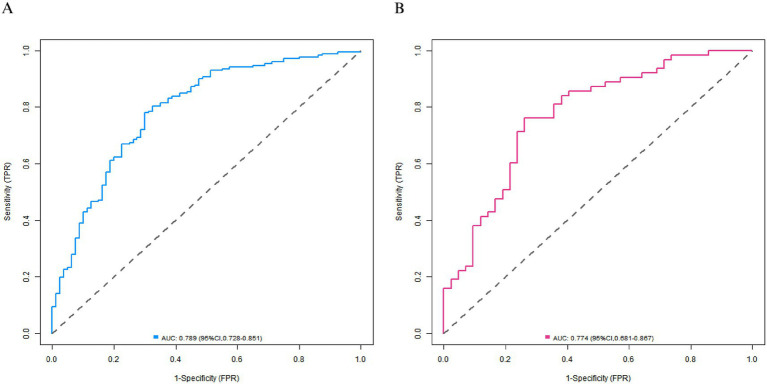
Receiver operating characteristic curves of patients with lower-limb motor dysfunction after stroke in the training set **(A)** and the validation set **(B)**.

In the training set, the C-index of the nomogram model was 0.792, and the *p* value of the Hosmer-Lemeshow test was 0.866, indicating a good fit of the model. Additionally, Bootstrap validation with 1,000 resamples yielded a median C-index of 0.785 (95% CI: 0.742–0.828), which further confirmed the model’s stability. The ROC curve showed that the AUC was 0.789 (95% CI: 0.728–0.851), with a sensitivity of 0.779 and a specificity of 0.700. In the validation set, the C-index was 0.778, the *p* value of the Hosmer-Lemeshow test was 0.442, the AUC was 0.774 (95% CI: 0.681–0.867), with a sensitivity of 0.714 and a specificity of 0.738. Compared with traditional single-indicator prediction, the nomogram model improved the C-index by 16.1% over using baseline FMA score alone (C-index = 0.682) in the training set, and by 20.6% over using age alone (C-index = 0.645) in the validation set. The calibration curve and ROC curve are shown in Figures 2, 3, respectively.

### Decision curve analysis

**Figure 4:**
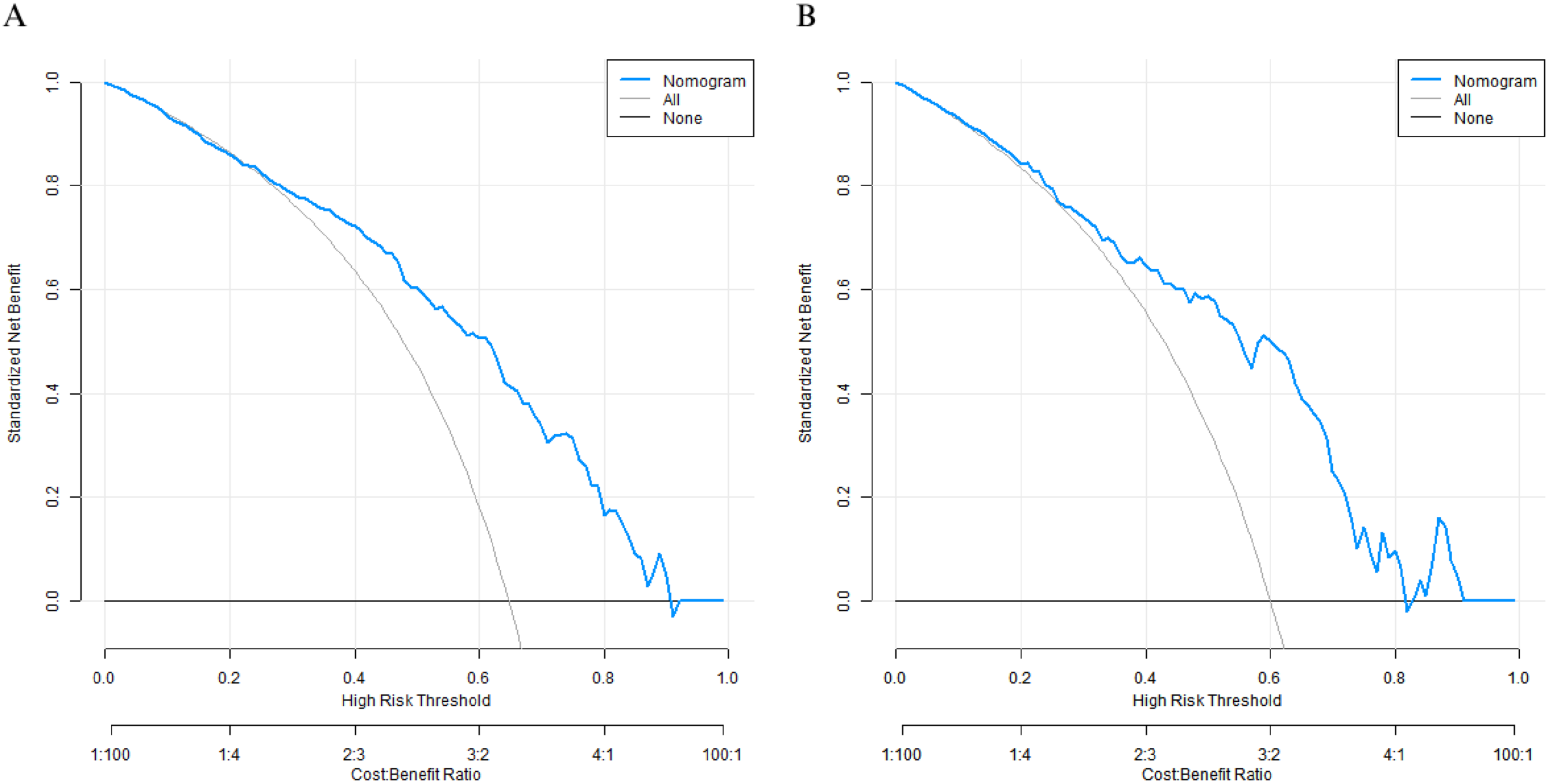
Decision curves in the training set **(A)** and the validation set **(B)**.

Decision curve analysis demonstrated that when the threshold probability ranged between 0.10 and 0.80, the application of the nomogram model in this study to predict the therapeutic efficacy of the conventional treatment regimen combined with functional electrical stimulation mirror therapy training for patients with lower limb motor dysfunction after cerebral infarction had the optimal net benefit (Figure 4).

## Discussion

In this study, we developed and validated a novel nomogram model to predict rehabilitation efficacy in 510 post-stroke patients with lower-limb motor dysfunction. The model incorporated six key predictors—age, baseline NIHSS/FMA scores, FES stimulation parameters (intensity/frequency), and mirror therapy duration—demonstrating robust discriminative ability (training set C-index: 0.792; validation set: 0.778) and calibration (Hosmer-Lemeshow *p* > 0.05). Notably, the model’s multi-dimensional integration (AUC: 0.789 (95% CI: 0.728–0.851) in training; AUC: 0.774 (95% CI:0.681–0.867) in validation) addresses critical limitations of conventional single-parameter assessments. Clinically, its visual scoring system and superior net benefit on decision curve analysis (threshold probability 10–80%) offer tangible advantages over traditional regression approaches for personalized rehabilitation planning.

Multivariate analysis showed that for every 1-year increase in age, the OR of treatment effectiveness decreased by 0.955 (95% CI: 0.924–0.987), suggesting that the efficacy is worse in elderly patients. However, this study did not collect data on pre-stroke exercise status (e.g., weekly exercise frequency, intensity), which may confound the association between age and efficacy; future studies should include this indicator. This may be related to the decline of neural plasticity, muscle atrophy, and the increase of comorbidities caused by aging (15). Studies have shown that the neuronal regeneration ability of elderly stroke patients is weakened, the efficiency of muscle contraction induced by FES stimulation is reduced, and the activation degree of the visual-motor cortex dependent on mirror therapy also decreases with age (16). In clinical practice, for elderly patients, it may be necessary to increase the FES stimulation intensity or extend the mirror training duration to make up for the age-related physiological disadvantages. For every 1-point increase in the baseline NIHSS score, the effective OR decreased by 0.908 (95% CI: 0.850–0.970), while for every 1-point increase in the baseline FMA score, the OR increased by 1.212 (95% CI: 1.122–1.309), reflecting the two-way influence of the degree of neurological deficit and baseline motor function (17). Patients with a higher NIHSS score have a wider range of brain damage, and it is more difficult to reshape the motor cortex; while a high baseline FMA score indicates better residual motor function and greater rehabilitation potential (18). This suggests that clinical baseline neurological function assessment should be used as the core indicator for efficacy prediction. For post-stroke patients with a high NIHSS score and a low FMA score, it is necessary to strengthen the intervention intensity of FES and mirror therapy on the basis of conventional treatment (19). The FES stimulation intensity (OR = 1.061 for every 1-mA increase) and frequency (OR = 1.125 for every 1-Hz increase) are both independent protective factors. The mechanism may be that an appropriate electrical stimulation intensity can effectively activate *α*-motor neurons, induce rhythmic muscle contraction, and enhance the strength of the quadriceps femoris muscle and the dorsiflexion function of the ankle joint; while an increase in frequency can improve the conduction efficiency of nerve impulses and promote the reconstruction of motor patterns (20). In this study, the stimulation intensity ranged from 10–40 mA, and the frequency was 20–50 Hz, which is consistent with the “optimal treatment window” in previous studies. However, individual tolerance differences should be noted to avoid muscle fatigue or skin damage caused by excessive intensity (21). For every 1-min increase in mirror therapy training duration, the effective OR increased by 1.054 (95% CI: 1.002–1.109), indicating a positive correlation between treatment dose and efficacy. Mirror therapy activates the primary motor cortex and secondary motor areas through visual feedback, promoting the reconstruction of motor imagery of the affected limb, and the accumulation of training duration can strengthen this neural remodeling effect (21). In this study, patients trained for 30 min each time, 1–2 times a day, which is in line with the standard intervention protocol of mirror therapy. However, in clinical practice, the duration can be adjusted according to the patient’s tolerance. Especially for patients with a short attention-maintaining time, segmented training can be used to ensure the total dose.

In this study, independent influencing factors were determined through Logistic regression, and the constructed nomogram model showed good prediction performance in both the training set and the validation set. The C-index exceeded 0.75, indicating that the model has a high degree of discrimination. The 7:3 split of training and validation sets is a standard ratio in nomogram development [consistent with previous studies (13)]. Although the validation set is smaller, baseline indicators (e.g., age, baseline FMA score, stroke type) were comparable between the two sets (all *p* > 0.05, Table 1), which minimized potential impacts on model calibration; the consistent C-index (training set: 0.792; validation set: 0.778) and similar AUC values further confirm the model’s stability across groups. From a clinical application perspective, this model can predict the efficacy before treatment through the patient’s age, baseline neurological function, and intervention parameters, providing a basis for formulating individualized treatment plans in practical scenarios: (1) In primary hospitals, it can help general practitioners quickly screen patients who may benefit less from standard FES-mirror therapy (predicted probability <30%) and refer them to tertiary hospitals for intensive intervention; (2) In rehabilitation centers, it can guide therapists to adjust treatment parameters (e.g., increasing FES intensity by 5–10 mA for patients with predicted probability 30–50%) instead of adopting a “one-size-fits-all” regimen; (3) For patients with predicted probability >70%, it can optimize resource allocation by reducing unnecessary follow-up frequency (from weekly to biweekly) to avoid over-intervention. The decision curve further confirms that the model has a net-benefit advantage at most threshold probabilities, which is more scientific than traditional empirical evaluations.

However, this study has the following limitations. First, all samples were from a single center. Although random grouping was used, multi-center external validation was not carried out, so it may be affected by regional medical levels or patient population characteristics. Second, the study included patients with a disease course ≤ 6 months and did not cover the chronic-phase population. The applicability of the model in long-term rehabilitation needs further verification. Third, subjective factors such as the patient’s psychological state (e.g., anxiety, depression) and social support (e.g., family care, community rehabilitation resources) were not included. These variables may affect the model’s predictive performance: for example, patients with severe anxiety may have lower compliance with mirror therapy (reducing actual training duration), leading to underestimated efficacy by the model; patients with sufficient social support may receive additional home training, resulting in overestimated efficacy. However, due to the lack of standardized assessment tools in the initial data collection stage, these variables could not be included, which is a limitation of this study. However, the advantages of this study are also significant: First, it is the first to integrate treatment variables such as FES stimulation parameters (intensity, frequency) and mirror therapy training duration, breaking through the traditional prediction model based only on baseline characteristics. Second, a nomogram visual model was used, which is easy to operate and convenient for clinicians to apply quickly. Finally, the sample size was relatively large, and double-validation was carried out through the training set and the validation set, enhancing the reliability of the model and providing a generalizable prediction tool for precise rehabilitation.

Future studies can be expanded in four aspects: First, multi-center, large-sample external validation should be carried out across different regions (e.g., north vs. south China) and hospital levels (primary vs. tertiary) to further evaluate the model’s applicability in diverse medical environments. Second, chronic-phase patients (disease duration >6 months) and more influencing factors (such as psychological state assessed by the Hospital Anxiety and Depression Scale [HADS], social support evaluated by the Multidimensional Scale of Perceived Social Support [MSPSS]) should be included to improve the prediction dimensions. Third, imaging biomarkers (such as motor cortex activation in fMRI, infarct penumbra volume in MRI) should be combined to explore the relationship between neural mechanisms and the prediction model, providing a more accurate theoretical basis for individualized rehabilitation. Fourth, as mentioned earlier, the efficacy of FES-mirror therapy should be compared with other visual feedback-based interventions to select optimal regimens for different patient subgroups.

In conclusion, the nomogram model constructed in this study integrates age, baseline NIHSS score, baseline FMA score, and treatment parameters of FES and mirror therapy, providing a visual tool for predicting the efficacy of patients with lower-limb motor dysfunction after cerebral infarction. The key indicators in the model not only reflect the patient’s baseline neurological function status but also embody the intervention dose effect of functional electrical stimulation and mirror therapy, which is in line with the interaction characteristics of neural plasticity and treatment intensity in stroke rehabilitation. Despite the limitations of single-center samples and the lack of external validation, it provides a quantitative basis for clinically screening patients with a low probability of efficacy at an early stage and dynamically adjusting rehabilitation plans. In the future, multi-center, large-sample studies are needed to further verify the efficacy of the model, and imaging indicators should be combined to deepen the exploration of the mechanism, promoting the standardized application of the prediction model in precise rehabilitation.

## Data Availability

The original contributions presented in the study are included in the article/supplementary material, further inquiries can be directed to the corresponding author.
